# Superficial angiomyxoma in a pregnant cow

**DOI:** 10.4102/jsava.v91i0.2004

**Published:** 2020-07-16

**Authors:** Annalisa Rizzo, Leonardo Della Salda, Mariarita Romanucci, Stefano Ciccarelli, Carmela Valastro, Michela Galgano, Raffaele L. Sciorsci

**Affiliations:** 1 Department of Veterinary Medicine, University of Bari Aldo Moro, Bari, Italy; 2 Faculty of Veterinary Medicine, University of Teramo, Teramo, Italy

**Keywords:** angiomyxoma, cow, cutaneous, histopathology, hormone dependency, immunohistochemistry, pregnancy

## Abstract

A 3-year-old, pregnant, Alpine Brown cow showed a rapidly growing, pedunculated, skin mass located at the umbilical region, reaching 8 kg in weight over a 3-month period after its initial detection. Six days after parturition, the mass was completely surgically excised. During the follow-up period, the cow remained in good health, without signs of recurrence, and showed increased milk production. Histological examination of the mass revealed a loose proliferation of spindle-shaped or stellate cells, immersed in an abundant myxoid matrix with admixed numerous thin-walled blood vessels. Immunohistochemically, the tumour cells were positive for vimentin, α-smooth muscle actin, and desmin. Gross and histopathological features were compatible with superficial angiomyxoma, a subtype of angiomyxoma rarely described in humans, but not in the veterinary literature. The tumour did not infiltrate into the surrounding tissues, and there was no post-excision recurrence after 3 months. The possibility of hormonal dependence of the tumour during pregnancy is discussed based on such findings in some human cases.

## Introduction

Tumour incidence in bovine species has increased over the last few years, and cattle have the second highest incidence of all tumours among domesticated animals after dogs (Marosfoi, Baba & Catoi 2009; Shruthi et al. 2018). Tumours affecting the skin at the dermal or subcutaneous level may be of mesenchymal origin, including fibroma/fibrosarcoma, haemangioma/haemangiosarcoma, lymphoma, and myxoma (Hendrick et al. 1998; Shruthi et al. 2018). Myxoma and myxosarcoma are tumours of fibroblastic origin distinguished by their abundant myxoid matrix rich in mucopolysaccharides (Hendrick 2017). In humans, myxomas arise in many different locations, including the skin, genitourinary tract, and heart (Allen 2000). The term angiomyxoma was chosen because of its similarity to myxoma and its significant vascular component. Angiomyxomas are classified as superficial or aggressive. The term aggressive was introduced to emphasise the strongly invasive behaviour at the local level and the high risk of recurrence, seen in approximately 72% of human cases, although distant metastasis is rare (Allen 2000).

In veterinary medicine, angiomyxoma is extremely rare, with only a few reported cases. A splenic angiomyxoma in a 13-year-old dog, as well as a benign renal angiomyxoma in an 11-year-old dog with paraneoplastic hypercalcaemia, have been described (Gajanayake et al. 2010; Lee et al. 2016a). In addition, a multifocal, multilobular, intra-abdominal peritoneal neoplasm with macroscopic and histopathological features corresponding to an aggressive angiomyxoma was reported in a 2-year-old East Flemish cow (Opsomer et al. 2001). This cow presented with vague symptoms including dullness, loss of weight, and diarrhoea. The diagnosis of aggressive angiomyxoma was made following necropsy.

Cytological smears of myxomatous tumours are often difficult to prepare because of the mucoid consistency of the tumour and poor cell adhesion to slides (Meuten 2017).

In humans, the treatment of choice is radical surgical excision of the lesion leaving healthy surgical margins, but this is not always possible as many of these lesions are large and infiltrate the surrounding soft tissue (Micci & Brandal 2007).

This report presents the case of a 3-year-old Alpine Brown cow with an exophytic pedunculated superficial subcutaneous mass in the umbilical area with characteristics attributable to aggressive angiomyxoma. It is also the first veterinary description of this neoplasm arising and growing aggressively during pregnancy and in which postpartum surgical resolution led to rapid recovery of the animal, suggesting an associated hormonal component.

## Case presentation

A 3-year-old, Alpine Brown cow, weighing approximately 400 kg, was referred to the Veterinary Mobile Clinic of the University of Bari ‘Aldo Moro’ (Italy) for a skin mass located in the umbilical region that had rapidly increased in size.

The cow lived in the province of Bari, on a breeding farm consisting of 60 lactating Alpine Brown cows in an intensive rearing system and was being fed hay, concentrates and minerals. Water was available *ad libitum*.

The patient history revealed that during the 6th month of pregnancy, a small skin nodule located in the umbilical region was noted by the owner. Given the concurrent pregnancy, the farm veterinarian decided to postpone surgical excision to the postpartum period. During the following 3 months, the mass enlarged sufficiently to cause discomfort to the cow, manifesting with reduced food intake and a reluctance to walk.

Calving was eutocic and the calf was in good health. The cow, however, showed low milk production and continued to display non-specific signs of illness, which worsened in the 6 days after delivery.

**FIGURE 1:**
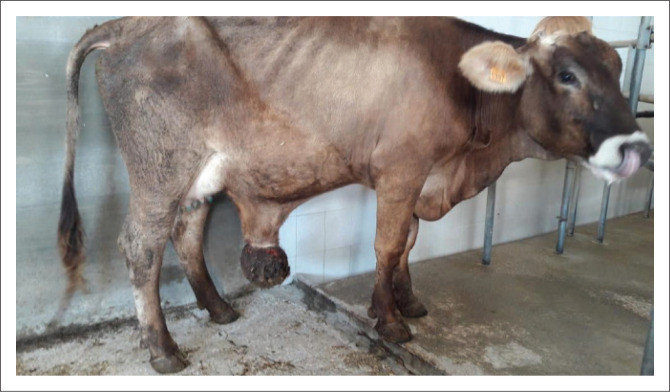
Cow at 6 days postpartum with poor body condition, showing a large skin mass located on the ventral abdomen in the umbilical region. The small udder is also visible.

On physical examination, the cow exhibited matted hair, a body condition score of 2.5/5, and a small udder. Rectal temperature was 38.7 °C and physiological organ functions were conserved (feeding, eating, urination, defaecation). On the ventral abdomen, a large, pedunculated, painless, soft to firm mass (30 cm × 25 cm) involving the skin and subcutaneous tissues of the umbilical region was detected (Figure 1). Multiple, small areas of superficial ulceration were noted on the skin covering the mass.

Preparation for surgical excision included sedation with intravenous xylazine hydrochloride (Nerfasin, Ati, Italy, 20 mg/mL) at a dose of 4 mg/100 kg (0.2 mL/100 kg), followed by epidural administration of 7 mL of procaine hydrochloride and adrenaline tartrate (Aticaina, Ati, Italy, procaine hydrochloride 40.0 mg/mL, adrenaline tartrate 0.036 mg/mL) between the L1 and L2 vertebral bodies. Immediately after the injection, the cow was placed in the right lateral recumbent position and restrained; its anterior and hind limbs were tied, for the safety of the operators.

After disinfection of the mass and its attachment area with alcohol and betadine, local regional anaesthesia was performed with procaine hydrochloride/adrenaline tartrate (Aticain, Ati, Italy, procaine hydrochloride 40.0 mg/mL, adrenaline tartrate 0.036 mg/mL), at 7 mL for each point. A diamond-shaped skin incision was made around the base of the mass. The pedunculated mass and subcutaneous tissue were detached following dissection using scissors with a rounded tip. The area was highly vascularised; therefore, numerous ligatures were placed to reduce bleeding. The mass appeared to be well circumscribed, without infiltration of the surrounding tissues of the abdominal wall, and complete surgical excision was performed. Subsequently, the skin and subcutaneous tissue were opposed using U-stitches with resorbable thread (Surgicryl USP 3-4).

**FIGURE 2:**
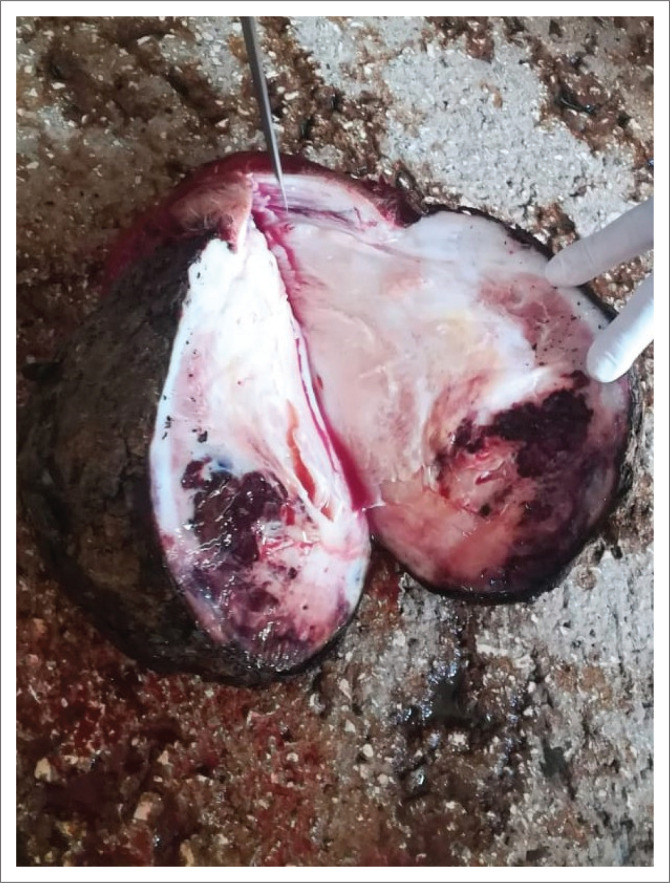
Cut section of the resected mass showing colour heterogeneity ranging from diffusely white to multifocally brownish areas and tinged with blood from haemorrhage in its central region.

The mass weighed 8 kg and was predominantly gelatinous and glistening when observed on the cut section, exhibiting colour heterogeneity ranging from diffusely white to multifocally brown and appearing tinged with blood from haemorrhage in its central region (Figure 2).

Multiple samples of the mass were fixed in 10% neutral buffered formalin, wax-embedded for histology, and 5-*μ*m thick sections were stained with haematoxylin and eosin. Additional sections of the mass were also stained with alcian blue at pH 2.5 and subjected to immunohistochemistry using primary antibodies directed against vimentin (1 in 100 dilution, V9, mouse monoclonal; DAKO), α-smooth muscle actin (α-SMA; 1 in 400 dilution, 1A4, mouse monoclonal; DAKO), desmin (1 in 50 dilution, D33, mouse monoclonal; DAKO), and von Willebrand factor (vWF; 1 in 400 dilution, rabbit polyclonal; DAKO), according to a previously-described technique (Romanucci et al. 2019). Immune complexes were treated with secondary biotinylated goat anti-mouse or anti-rabbit antibody (1 in 200 dilution; Vector Laboratories, Inc., Burlingame, CA) and subsequently detected using an avidin-biotin complex (ABC) method (Vectastain® ABC Kit; Vector Laboratories, Inc.). Peroxidase activity was detected using 0.1% hydrogen peroxide in 3,3′-diaminobenzidine solution (Sigma–Aldrich, Saint Louis, MO). Sections were finally counterstained with Mayer’s haematoxylin (Merck, Darmstadt, Germany). Sections of canine and bovine small intestine and skeletal muscle (for vimentin, α-SMA, and desmin), as well as vWF-positive canine hemangiosarcoma, were used as positive controls.

**FIGURE 3:**
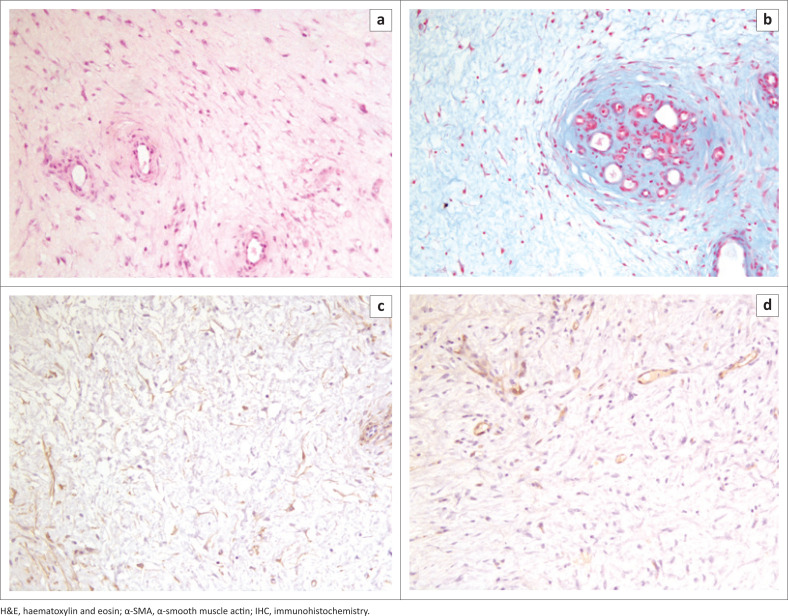
(a) Histological features of the mass consisting of a loose neoplastic proliferation of spindle-shaped or stellate cells, immersed in an abundant myxoid matrix with scattered thin-walled blood vessels. H&E, 20×. (b) Abundant myxomatous extracellular matrix positively stained with alcian blue. 20×. (c) Variably intense positivity of neoplastic cells for α-SMA. IHC, 20×. (d) Several von Willebrand factor-positive blood vessels admixed with neoplastic cells. IHC, 20×.

Histological examination of the mass revealed a loose proliferation of spindle-shaped or stellate cells with small spindle-shaped hyperchromatic nuclei, immersed in an abundant amorphous myxoid matrix with numerous admixed thin-walled blood vessels (Figure 3a). Anisocytosis, anisokaryosis, and cellular pleomorphism were mild, and rare mitotic figures were detected. A myxomatous extracellular matrix was positively stained with alcian blue (Figure 3b). Multifocal, low to moderate infiltration of neutrophils and lymphocytes located around blood vessels or interspersed between neoplastic cells were also observed. In addition, multifocal areas of haemorrhage and necrosis were found within the mass. Immunohistochemically, tumour cells were intensely and diffusely positive for vimentin, as expected for a tumour of mesenchymal tissue origin, and variably positive for α-SMA (Figure 3c) and desmin. Numerous blood vessels were also detected throughout the tumour as highlighted by positive endothelial labelling with von Willebrand factor (Figure 3d).

Gross and histopathological findings, immunohistochemical results, and similarity to previously-reported cases in humans (Abarzúa-Araya et al. 2016; Lee et al. 2016b; Okada et al. 2005) supported a diagnosis of superficial angiomyxoma.

During the follow-up period (at 7, 15, and 30 days and 3 months after surgery), the cow was in good health, without evidence of tumour recurrence, and had a normal appetite and increased milk production.

### Ethical consideration

I confirm that ethical clearance was not needed/required for the study.

## Discussion

When considering a diagnosis of angiomyxoma, the differential diagnoses include an inflammatory myofibroblastic tumour, which may display a myxoid/vascular pattern. An inflammatory myofibroblastic tumour, however, is usually characterised by a haphazard fascicular arrangement of plump spindle cells, with more varied cellularity, including hypocellular areas, and more dense inflammation, including plasma cells (Alderman & Kunju 2014). In contrast to myxoma, myxoid peripheral nerve sheath tumours have a multilobular configuration and the tumour cells are arranged in concentric whorls or form palisades (Gross et al. 2005). Bovine fibropapilloma is typically characterised by a dermal proliferation of large, plump fibroblasts haphazardly arranged in whorls and fascicles (Mauldin & Peters-Kennedy 2016). Similarly, a skin tag (fibroepithelial polyp, acrochordon) was excluded, since it is typically a lesion consisting of redundant mature dermal fibrous tissue (Goldschmidt & Goldschmidt 2017). Exuberant granulation tissue, which is the result of a reparative process characterised by neovascularisation and a proliferation of fibroblasts within a proteoglycan rich matrix (Mauldin & Peters-Kennedy 2016) was also excluded, since the mass was only superficially ulcerated, and cutaneous ulceration occurred later as a consequence of progressive growth of the mass and superficial trauma.

Immunohistochemically, neoplastic cells of angiomyxoma often display variable positivity for α-SMA and desmin (Gajanayake et al. 2010; Lee et al. 2016a; Okada et al. 2005; Shaver, Kolker & Bennett 2018), and a myofibroblastic phenotype has been hypothesised (Gajanayake et al. 2010). Regarding the intensity of alcian blue staining, the intercellular matrix may stain weakly in angiomyxoma, in contrast to the dense positive staining usually observable in myxoma due to the presence of hyaluronic acid (Bedir, Yılmaz & Calapoğlu 2018). Additionally, in humans, neutrophilic infiltration not associated with necrosis is considered a unique histologic feature of superficial angiomyxoma, distinguishing it from other lesions with a myxoid component and representing an important histologic clue for the differential diagnosis. The pathogenesis of this neutrophilic infiltration remains unknown (Okada et al. 2005).

The presence of multiple areas of haemorrhage and necrosis within angiomyxomatous tissue is also not uncommon (Gajanayake et al. 2010; Okada et al. 2005; Opsomer et al. 2001).

Although angiomyxoma is usually a slow growing tumour, an increased rate of growth during pregnancy has been described in human literature (Zangmo et al. 2016), which could be related to a hormone dependency (Orfanelli et al. 2016). Orfanelli et al. (2016) reported on a primigravid woman who had two vulval labial angiomyxomas which developed during pregnancy, the larger of which was surgically removed during a caesarean section; the second smaller mass, however, resolved spontaneously 2 weeks postpartum. The tumour cells of the removed mass were immunohistochemically positive for CD34, oestrogen, and progesterone receptors (Orfanelli et al. 2016). In the present case, the rapid growth of the mass could be speculated to be related to the pregnancy of the cow. An immunohistochemical investigation of hormonal markers would have been interesting in the bovine tumour reported here; however, this was not performed.

In conclusion, to the best of the authors’ knowledge, this is the first description in the veterinary literature of a cutaneous neoplasm with gross, histopathological, and immunohistochemical features typical of a superficial aggressive angiomyxoma, for which complete surgical excision was successfully performed, leading to rapid recovery of the animal. Other interesting features of this case were the superficial cutaneous occurrence and rapid growth during the pregnancy of the cow, apparent suppression of lactation until the tumour was excised, and the systemic effect it had on the condition and habitus of the cow, suggesting a possible hormonal component, as has been demonstrated using hormone markers in some aggressive angiomyxomas in humans.
